# Inflammatory pseudotumour: A rare tumor of lung

**DOI:** 10.1016/j.amsu.2018.10.033

**Published:** 2018-11-03

**Authors:** Nisha Marwah, Namita Bhutani, Sakshi Dahiya, Rajeev Sen

**Affiliations:** Dept. of Pathology, PGIMS, Rohtak, Haryana, India

**Keywords:** Benign tumor, Case report, Chest radiograph, Inflammatory myofibroblastic tumor, Lung

## Abstract

Inflammatory pseudotumours of the lungs have rarely been reported. These have been described as a benign entity of unknown origin and are often locally invasive requiring extensive pulmonary resection. Complete resection is the key to prevent recurrence and the prognosis is excellent following surgery. We describe a patient with inflammatory pseudotumour of the lung. He was a middle aged man who presented with haemotysis and the chest X-ray and computerized tomography were indicative of a nonbenign lesion in the right upper lobe. Excision biopsy confirmed the diagnosis of inflammatory myofibroblastic pseudotumour of the lung. This is a rare inflammatory nonneoplastic condition commonly affecting children and young adults.

## Highlights

• The inflammatory pseudotumor of the lung is a rare entity.
• Surgical excision is usually indicated to reach a firm diagnosis and cure.
• As preoperative investigation is not diagnostic, excision of the mass is imperative in order to exclude malignancy.
• Despite being a benign lesion, its potential for recurrence and local invasion requires complete surgical resection.
• Complete resection, when possible, is safe and leads to excellent survival and remains the key to prevent recurrence.

## 1 Introduction

Inflammatory myofibroblastic tumor (IMT) is a rare benign tumor, accounting for 0.7% of all lung tumors [1,2]. First described by Brunn in 1939, it is an inflammatory, reactive, and nonneoplastic process characterized by unregulated growth of inflammatory cells [3]. It occurs most commonly in children and young adults and is usually found incidentally. The origin is unknown, but recent studies have shown that it is a true tumor rather than a reaction process [4]. The clinical and radiological manifestations are diverse and nonspecific. Diagnosis is difficult to establish without surgical resection [2,5]. Since inflammatory pseudotumor (IPT) is a benign disease which ambiguously presents with a tumor-like shadow having an irregular periphery on imaging, a diagnosis discriminating between IPT and malignant tumor is essential. In addition, the natural history and appropriate treatment of this disease remain unclear. However, there is a discrepancy between the pathologic findings and clinical behavior, including a recurrence and invasion to the adjacent organs. Such inexplicable characteristics prevent the surgeon from clearly identifying the disease. These are often locally invasive requiring extensive pulmonary resection [6]. Complete resection is advocated to prevent local recurrence and leads to excellent survival. The SCARE criteria were utilized for this case report [7].

### 1.1 Case report

**Fig. 1:**
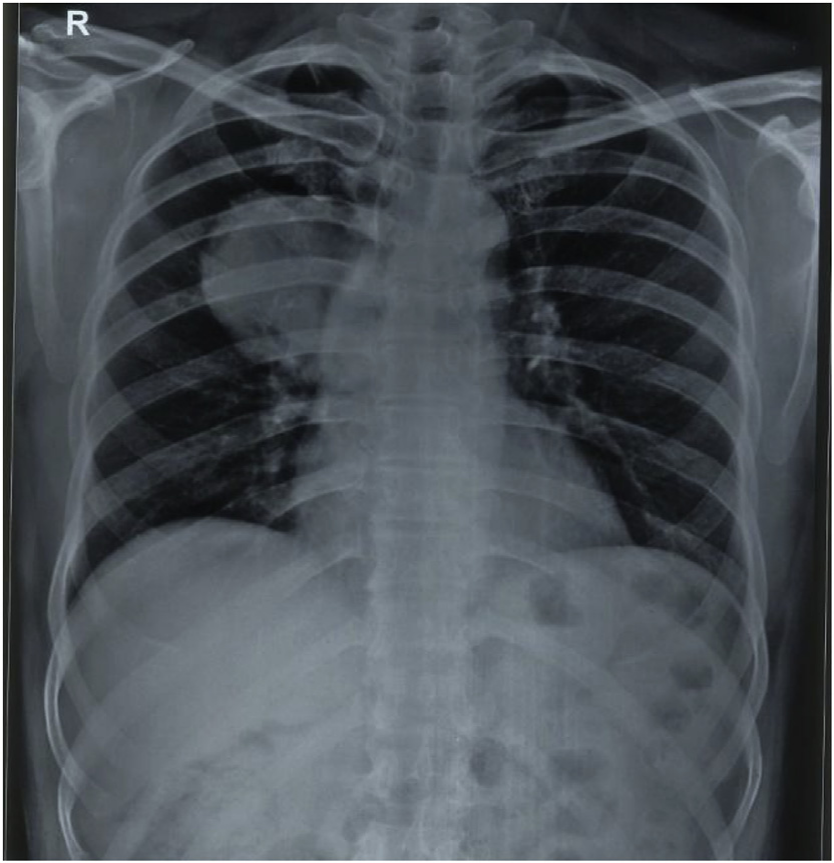
Chest x-ray showing growth in right upper lobe.

**Fig. 2:**
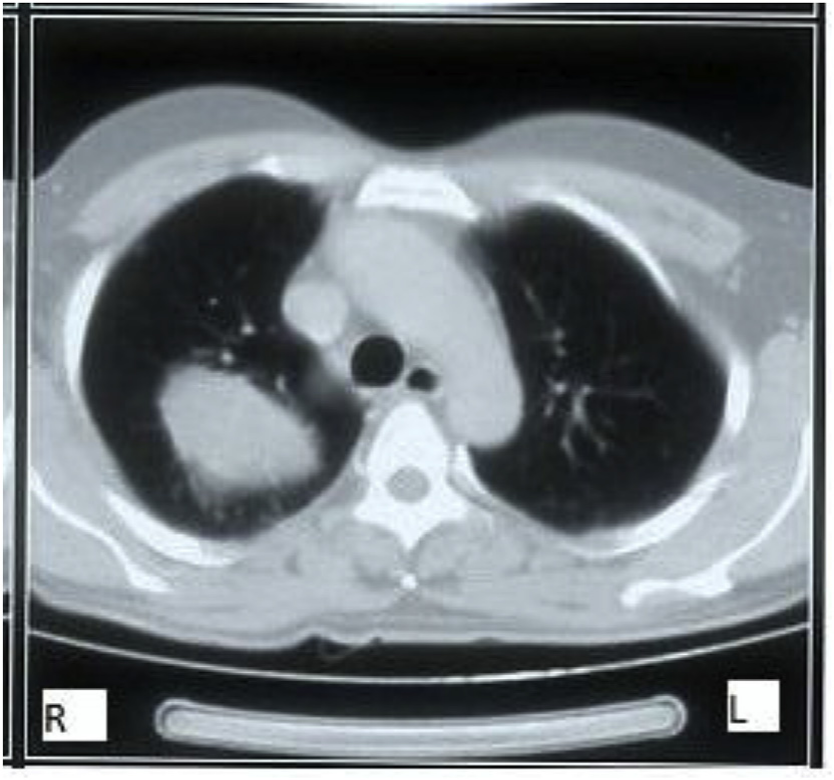
CT scan chest showing a heterogenously enhancing lesion in right upper lobe.

**Fig. 3:**
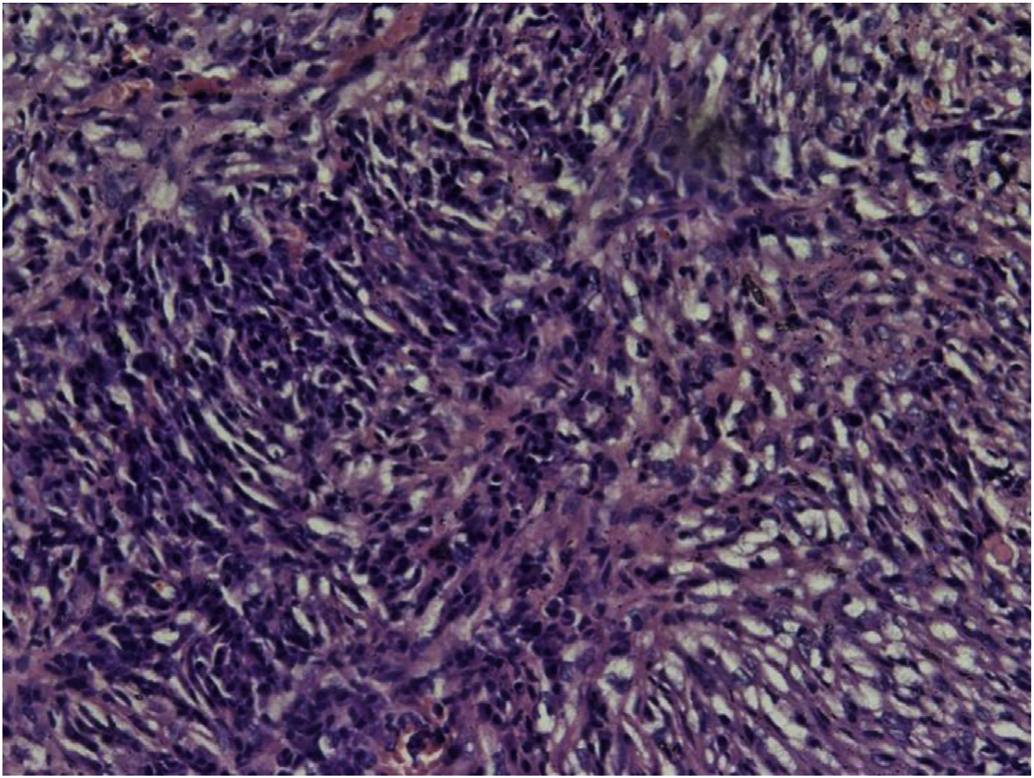
ON H&E: Inflammatory myofibroblastic tumor (100 X).

**Fig. 4:**
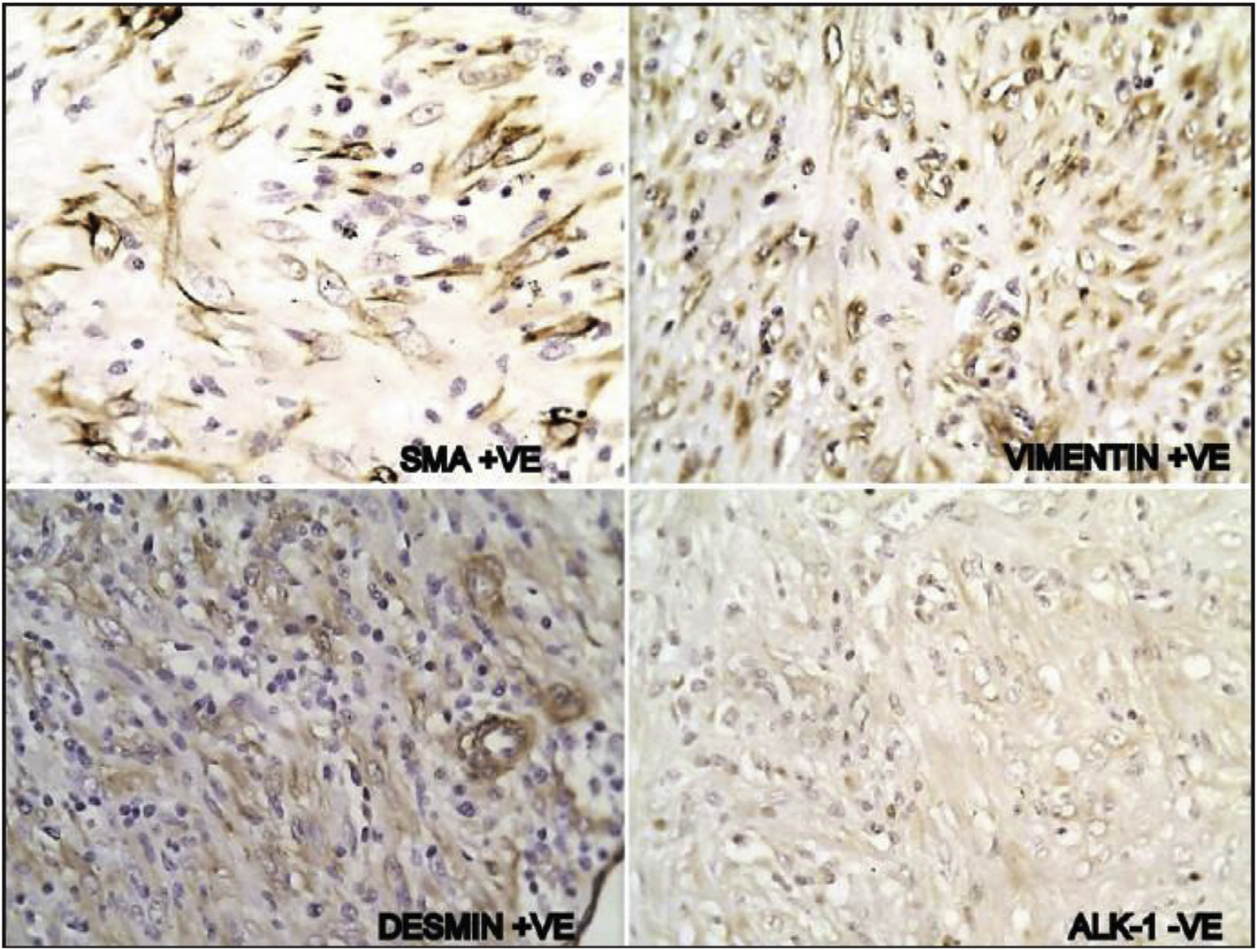
Immunohistochemistry (200 X).

A 40-years-old male non-smoker presented to chest outpatient department with complaints of recurrent mild haemoptysis for 2 months, which was progressive in nature. Chest radiograph revealed a 2.5 × 2 cm lesion in the posterior segment of the right upper lobe of the lung (Fig. 1). The medical history was noncontributory. A computed tomographic (CT) scan of the chest confirmed the chest radiograph findings; a solid mass was noted in the posterior segment of the right upper lobe of the lung (Fig. 2). There was no hilar lymphadenopathy. Sputum microscopy, culture, and cytological examination were essentially normal. The ESR was 18, the haemoglobin 15.5 g/dL, and the leukocyte count 9.6 × 10^9^/L. The other serum haematological and biochemical results were normal. In view of the patient's ongoing haemoptysis and lack of response to antibiotics he underwent bronchoscopy which revealed a growth in right upper lobe with endobronchial obstruction. At the same time endobronchial biopsy was taken which was sent for histopathological examination. Microscopically, the biopsy showed a heavy inflammatory cell infiltrate composed predominantly of lymphocytes, with plasma cells and histiocytes. Foamy histiocytes with macrophages were also seen, as well as occasional eosinophils and neutrophils. Focal areas of micro-abscess formation with necrosis were also noted. A marked degree of fibrosis was present with proliferating myofibroblasts. The histological characteristics were compatible with an inflammatory myofibroblastic pseudotumour (Fig. 3). On immunohistochemistry, vimentin, SMA, ALK-1 and desmin were positive, further corroborating the diagnosis (Fig. 4). Surgery, for diagnostic and therapeutic purposes, consisted of a right pneumonectomy. The postoperative course was uneventful, the patient was discharged from the hospital one week later and his symptoms improved.

## 2 Discussion

Inflammatory pseudotumors of the lung are rare and were first described in the lung in 1939 [1]. They are not limited to the lung and can grow in any organ such as the brain, liver, spleen, lymph nodes, salivary glands, breast, soft tissues and skin [8]. Although they are regarded as inflammatory or reactive lesions rather than neoplasms, they may have features such as local invasion or recurrence, distant metastases, and cytogenetic clonal changes [9]. Inflammatory pseudotumors are usually considered to be benign tumors, principally occurring in younger patients. They are the most common in children younger than 16 years. More than half of patients are less than 40 years of age [10].

The aetiology and pathogenesis remain uncertain. There are several theories, most of which postulate an exaggerated immunologic response to a viral or foreign antigen antibody reaction [4]. They typically consist of variable amounts of stromal and cellular elements, with the myofibroblast, a cell involved in tissue repair, recognized as the principal cell-type [11]. Depending on the major histopathologic features, inflammatory pseudotumors are divided into the following types: fibrous histiocytoma, lymphoplasmacytic, and organising pneumonia. Because of their variable histology, these masses have several synonyms, including plasma cell granuloma, myofibroblastic tumor, xanthoma, xanthogranuloma, xanthomatous pseudotumour, and plasma cell histiocytoma [8].

The neoplastic subset of IMT usually shows evidence of clonal cytogenetic abnormalities involving 2p23 that encodes ALK gene, and typically occurs in children and young adults [12]. Those that are not neoplastic are distinguished by the generally older age of the patients and the ill-defined or irregular contour of the lesion due to a prominent organising pneumonia component and fibrosis at the edge of the tumor. Inflammatory pseudotumors (IPT) in different age groups or in different locations represent diverse clinicopathologic entities. But, IPT occurring in the pediatric age group is most often a neoplasm with frequent ALK positivity and might properly be referred to as IMT, IPT in adults may include more nonneoplastic processes with much less frequent ALK positivity [13]. Inflammatory pseudotumor in the lower urogenital tract has been postulated to be a reparative process with a morphologic resemblance to nodular fasciitis. In IMT, ALK immunopositivity is a highly sensitive and specific indicator of a 2p23 abnormality and is thus a useful surrogate for molecular or cytogenetic testing. Overall sensitivity of ALK in IPT is approximately 50%, although it varies greatly depending on the age of patients.

Many patients are asymptomatic, with inflammatory pseudotumor discovered incidentally on chest radiograph [14]. If the patients are symptomatic, cough, fever, weight loss, fatigue, haemoptysis, dyspnoea, clubbing, chest pain, and arthralgias may be noted [7,14]. No specific findings on physical or laboratory examinations exist. Radiological examination usually demonstrates a solitary peripheral nodule or mass of 1–10 cm in diameter and the lesions are typically peripheral and in the lower lobes [15]. Radiographic images and invasive diagnostic procedures, including bronchoscopy and percutaneous fine needle aspiration biopsy, are considered insufficient for diagnosis. The most important radiographic differential diagnosis in adults with IPT is primary lung cancer. Consequently, surgery is crucial for both diagnostic and therapeutic reasons [14].

Grossly, IMFT are well circumscribed, non-encapsulated, firm, usually yellow-white masses containing variable inflammation, haemorrhage, calcification, and rarely cavitation. Most are parenchymal but some are endobronchial and may cause airway obstruction. Less than 5% invade the mediastinum and/or chest wall. Local recurrence is attributed to incomplete resection of the primary lesion. Metastasis of the tumor to mediastinum or the brain even many years after complete resection has been described. Rarely, simultaneous intra- and extrathoracic locations may occur.

Microscopically, the lesions consist of variable mixtures of fibroblasts and granulation tissue, fibrous tissue, and inflammatory cells including lymphocytes, histiocytes, giant cells, macrophages, neutrophils, eosinophils, and typically large numbers of plasma cells [1,3,4]. The differential diagnosis can be quite wide, specific considerations include solitary metastases from an osteosarcoma or mucinous tumor, intra-thoracic soft tissue sarcoma and pulmonary hamartoma [14,16].

Complete surgical resection remains the best treatment, to exclude malignancy and to achieve cure [16,17]. Wedge resection is adequate treatment if removal is complete. Lobectomy should be performed if it is required for complete resection and if the patient's pulmonary reserve is adequate. Non-surgical treatment modalities including radiotherapy, chemotherapy, and corticosteroids may have a place in the setting of incomplete surgical resection, multifocal disease, postoperative tumor recurrence, or contraindications to lung resection [[16], [17], [18]]. Corticosteroids are generally not useful in adults, although good results have been reported in children in cases of unresectable tumors or hilar and mediastinal invasion. Chemotherapy is useful in cases of multifocal, invasive lesions or in cases of local recurrence [8]. Although spontaneous regression may occur, local expansion may cause significant morbidity and occasional death. The prognosis of these rare tumors is excellent after complete surgical excision. There is a low incidence of recurrence with long-term follow up after complete removal of the mass [16,17]. Patients with recurrent disease should undergo re-resection [14,16].

## 3 Conclusion

Although inflammatory pseudotumours of the lungs are rare and the most common clinical picture is one of an asymptomatic, well-circumscribed lung mass that mimics cancer, clinicians need to bear in mind the diverse clinical presentations. Surgical excision is usually indicated to reach a firm diagnosis and cure. As preoperative investigation is not diagnostic, excision of the mass is imperative in order to exclude malignancy. Despite being a benign lesion, its potential for recurrence and local invasion requires complete surgical resection. Complete resection, when possible, is safe and leads to excellent survival and remains the key to prevent recurrence.

## Consent

Written informed consent was obtained from the patient for publication of this case series and any accompanying images.

We declare that there are no conflicts of interest amongst the authors.

We also state that there was no source of funding for this study.

## Provenance and peer review

Not commissioned, externally peer reviewed.

## Conflicts of interest

There is no conflict of interest.

## Funding

There is no source of funding.

## Ethical approval

Not applicable.

## Research registration unique identifying number (UIN)

Not applicable.

## Trial registry number

NA.

## Author contribution

**Nisha Marwah**-Designed the study.

**Namita Bhutani**-Collected the data and wrote the paper.

**Sakshi Dahiya**-Helped in data collection.

**Rajeev Sen**-Analysed the data.

## Guarator

Dr Pradeep Kajal.

## Appendix A Supplementary data

Scare Checklist Scare Checklist
